# Pyrvinium, a Potent Small Molecule Wnt Inhibitor, Promotes Wound Repair and Post-MI Cardiac Remodeling

**DOI:** 10.1371/journal.pone.0015521

**Published:** 2010-11-29

**Authors:** Sarika Saraswati, Maria P. Alfaro, Curtis A. Thorne, James Atkinson, Ethan Lee, Pampee P. Young

**Affiliations:** 1 Department of Pathology, Vanderbilt University Medical Center, Nashville, Tennessee, United States of America; 2 Department of Cell and Developmental Biology, Vanderbilt University Medical Center, Nashville, Tennessee, United States of America; 3 The Department of Veterans Affairs Medical Center, Nashville, Tennessee, United States of America; 4 Department of Internal Medicine, Vanderbilt University Medical Center, Nashville, Tennessee, United States of America; Biological Research Center of the Hungarian Academy of Sciences, Hungary

## Abstract

Wnt signaling plays an important role in developmental and stem cell biology. To test the hypothesis that temporary inhibition of Wnt signaling will enhance granulation tissue and promote angiogenesis in tissue repair, we employed a recently characterized small molecule Wnt inhibitor. Pyrvinium is an FDA-approved drug that we identified as a Wnt inhibitor in a chemical screen for small molecules that stabilize β-catenin and inhibit Axin degradation. Our subsequent characterization of pyrvinium has revealed that its critical cellular target in the Wnt pathway is Casein Kinase 1α. Daily administration of pyrvinium directly into polyvinyl alcohol (PVA) sponges implanted subcutaneously in mice generated better organized and vascularized granulation tissue; this compound also increased the proliferative index of the tissue within the sponges. To evaluate its effect in myocardial repair, we induced a myocardial infarction (MI) by coronary artery ligation and administered a single intramyocardial dose of pyrvinium. Mice were evaluated by echocardiography at 7 and 30 days post-MI and treatment; post mortem hearts were evaluated by histology at 30 days. Pyrvinium reduced adverse cardiac remodeling demonstrated by decreased left ventricular internal diameter in diastole (LVIDD) as compared to a control compound. Increased Ki-67^+^ cells were observed in peri-infarct and distal myocardium of pyrvinium-treated animals. These results need to be further followed-up to determine if therapeutic inhibition of canonical Wnt may avert adverse remodeling after ischemic injury and its impact on myocardial repair and regeneration.

## Introduction

Myocardial infarction (MI) is the leading cause of disability and death in the United States [1]. MI induces cardiomyocyte death and an inflammatory response that is followed by the formation of granulation tissue which results in scar formation [2]. The infarct injury affects the heart in a global manner and incites a process termed “ventricular remodeling” that affects the size, shape, and function of the heart and ultimately leads to organ dysfunction [2]. The decline in left ventricular function and adverse remodeling of the heart typically result in the progression of heart failure. Current therapies have limited effectiveness on adverse ventricular remodeling [3].

The non-canonical and canonical Wnt signaling pathways are indispensible for heart development [4], [5] and other biological processes including cell migration, cell proliferation, development [6], [7], and stem cell self-renewal [8], [9]. The central player of the “canonical” pathway is β-catenin, which is maintained at a low level in the cytoplasm by its association with a destruction complex. In the absence of Wnt signaling a β-catenin destruction complex that includes the tumor suppressors adenomatous polypolis coli (APC) and axin2, interacts with β-catenin [10] inside the cell. β-catenin gets phosphorylated at serine/threonine residues 33, 37 and 41 by Casein Kinase-1 and Glycogen Synthase Kinase-3β (GSK-3β). This results in the recruitment of a β-TrCP-containing E3 ubiquitin ligase that targets β-catenin for proteosomal degradation [11]. However, when the canonical Wnt signaling is on, the Wnt ligand binds to the frizzled receptor and LRP co-receptors [12]. This interaction recruits axin2 and disheveled to the LRP protein and frizzled receptor, respectively [12], [13], [14], and further inhibits the kinase activity of the β-catenin destruction complex [15]. Consequently, β-catenin accumulates and mobilizes into the nucleus where it interacts with the DNA-binding proteins of the Tcf/Lef family of high mobility group (HMG)-box proteins [8], [16]. The nuclear Tcf/Lef/β-catenin complex binds to the DNA and activates the transcription of Wnt target genes [17], [18].

Wnt signaling is quiescent in the adult heart [19]. A recent study [20], [21] in abstract form using Wnt (axin2-LacZ) reporter mice demonstrated that Wnt signaling is increased post-MI in the cardiomyocytes of the border zone and remote area between 7–21 days after infarct whereas infiltrating CD45^+^ inflammatory cells showed Wnt activation between 3–7 days post infarct [22]. Hence, endogenous activation of the Wnt pathway occurs in the heart in cardiomyocytes and other cells and is evident just prior to the initiation of the remodeling phase (day 10–26) of murine infarct repair.

Several genetic models suggest that Wnt inhibition may reduce adverse remodeling post injury. Transgenic mice in which β-catenin was downregulated in an α-MHC-restricted manner (*i.e.* resulting in lower cardiac Wnt signaling) demonstrated favorable ischemic remodeling [23]. Mice lacking Dishevelled-1 (resulting in attenuated Wnt signaling) exhibited decreased hypertrophic response after pressure overload induced by aortic banding [24]. Other groups reported functional deterioration after injury in mice expressing a stabilized β-catenin (*i.e.* activated Wnt signaling) in cardiomyocytes [25], [26]. We and others have shown that mesenchymal stem cells overexpressing sFRP2, a Wnt inhibitor, reduced cardiomyocyte apoptosis [27], [28]. Taken together, these studies suggest a role for Wnt inhibition in preventing maladaptive cardiac remodeling as well as in improved cardiac function after injury.

Although transgenic animal models of cardiac injury suggest a role for Wnt inhibition on cardiac repair and regeneration, pharmacological tools to inhibit Wnt signaling in a cardiac injury model have not been studied so far. In the present study we utilized a FDA-approved small molecule inhibitor, pyrvinium, which inhibits Wnt signaling by directly acting on a downstream Wnt signaling molecule Casein Kinase-1 to evaluate its therapeutic effect on wound and cardiac repair and regeneration.

## Results

### Inhibition of Wnt signaling by pyrvinium

**Figure 1:**
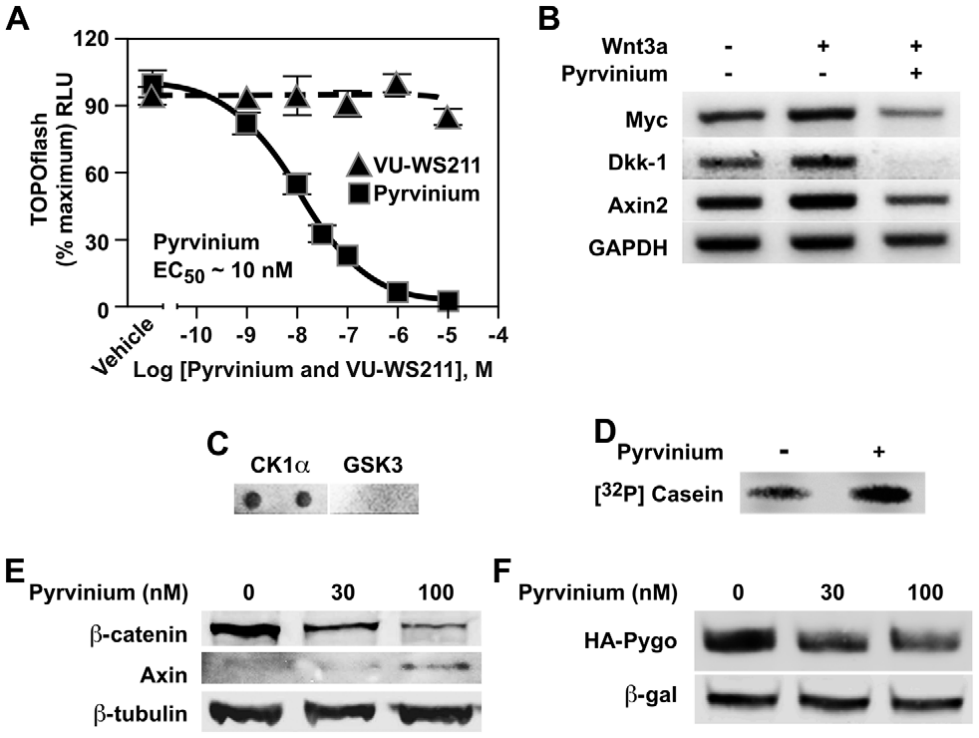
Pyrvinium inhibits Wnt signaling. (**A**) Pyrvinium inhibits TOPflash activation with an EC_50_ of ∼10 nM in contrast to the structurally related compound VU-WS211. HEK 293 STF (TOPflash) luciferase reporter cells incubated with Wnt3a-conditioned media were treated as indicated. Graph represents mean ± SEM of TOPflash signal normalized to cell number (performed in quadruplicate). (**B**) Pyrvinium (100 nM) decreases transcript levels of endogenous Wnt target genes Myc, Dkk-1, and Axin2 as assessed by real-time PCR. GAPDH is control. (**C**) Pyrvinium binds CK1α *in vitro*. CK1α and GSK3 (0.5 µg each, in duplicates) were spotted on nitrocellulose, incubated with pyrvinium (10 nM), and bound pyrvinium detected based on its fluorescent property. (**D**) Pyrvinium stimulates CK1α activity. CK1α (100 nM) was incubated with recombinant casein (100 nM) plus or minus pyrvinium (10 nM) in a kinase reaction containing [γ^32^P]ATP followed by SDS-PAGE and exposure to PhosphoImager screen. (**E**) Pyrvinium decreases and increases intracellular β-catenin and Axin levels, respectively. HEK 293 cells were treated for 16 hours as indicated, and cytoplasmic preparations were immunoblotted for β-catenin and Axin. β-tubulin is loading control. (**F**) Pyrvinium decrease steady-state levels of Pygopus. HEK 293 STF cells expressing HA-tagged human Pygopus-2 were treated with pyrvinium as indicated. Lysates were immunoblotted for HA. β-galactosidase (β-gal) is transfection control.

We previously developed a biochemical assay using *Xenopus laevis* egg extract that recapitulates Axin and β-catenin turnover in response to addition of recombinant Wnt co-receptor (LRP6) [29]. Recombinant LRP6 inhibits β-catenin degradation and stimulates Axin degradation in Xenopus extract. Using a system in which β-catenin is fused to firefly luciferase and Axin is fused to *Renilla* luciferase, we performed a high-throughput screen to identify small molecules that reverse the effects of recombinant LRP6. From this screen, we identified a FDA-approved antihelminthic compound (pyrvinium) that potently inhibits Wnt signaling in cultured mammalian cells. Pyrvinium inhibited Wnt-mediated transcription with an EC_50_ of ∼10 nM in contrast to a structurally related compound (VU-WS211), demonstrated by a luciferase-based reporter containing TCF/LEF binding sites (TOPflash) stably transfected in HEK 293 cells (HEK 293 STF) [30] (Figure 1A). Real-time RT-PCR identified inhibition of endogenous Wnt target genes Myc, Dkk-1, and Axin2 in the presence of pyrvinium (Figure 1B and Figure S1D, E, and F), consistent with the effect of pyrvinium on the TOPflash reporter. Based on *in vitro* reconstitution studies with purified proteins encoding known Wnt components, we found that the target of pyrvinium is Casein Kinase 1α (CK1α). Specificity of pyrvinium binding towards CK1α was demonstrated in a ligand-binding assay. Pyrvinium has an intrinsic fluorescent signal. CK1α and GSK3 were dotted on nitrocellulose and only the nitrocellulose dotted with CK1alpha showed pyrvinium fluorescence (Figure 1C). To assess the effect of pyrvinium on CK1α kinase activity, recombinant CK1α was incubated with [γ^32^P]ATP and casein in the presence or absence of pyrvinium. Phosphorylation of casein by CK1α was enhanced in the presence of pyrvinium indicated that pyrvinium activated CK1α kinase activity (Figure 1D). The predominant cytoplasmic effects of CK1α activation by pyrvinium in the Wnt pathway were to promote degradation of β-catenin and to inhibit Axin degradation. Indeed, immunoblot analysis of cytoplasmic preparations of HEK293 cells treated with increasing concentrations of pyrvinium demonstrated dose-dependent decreased and increased levels of β-catenin and Axin, respectively (Figure 1E and Figure S1A and B). Moreover, in the nucleus, pyrvinium promoted the degradation of Pygopus, a nuclear factor associated with the activation of a Wnt transcriptional program (Figure 1F and Figure S1C). Taken together, these results demonstrate that pyrvinium inhibits Wnt signaling. A detailed description of our identification of pyrvinium and the characterization of its mechanism of action will be presented elsewhere [31].

### Pyrvinium increases granulation tissue organization, proliferation, and vascularity in the sponge model of tissue repair

**Figure 2:**
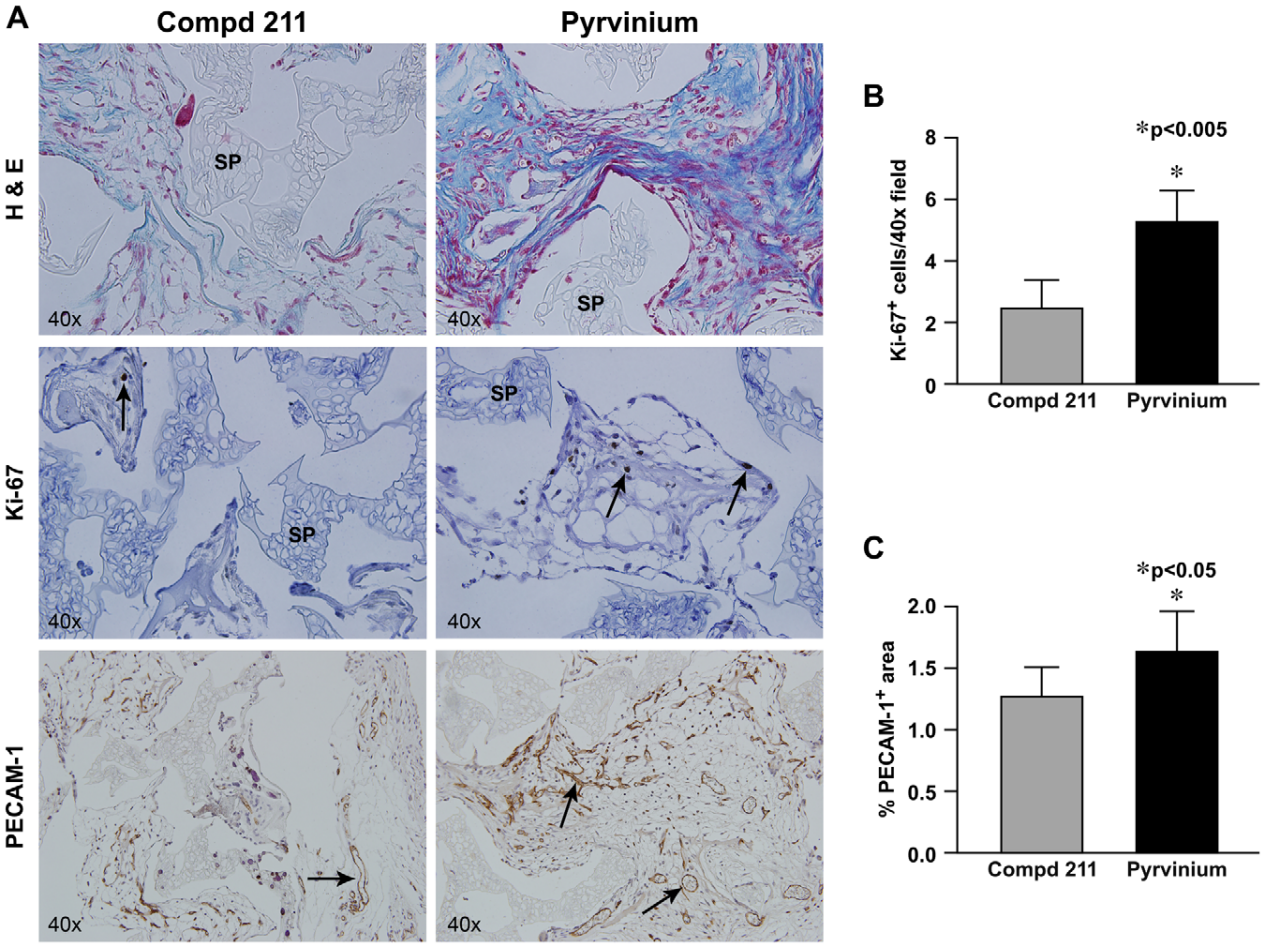
Pyrvinium increases granulation tissue organization, proliferation, and vascularity in the sponge model of tissue repair. (**A**) Representative images of the pyrvinium- and compd 211-treated sponges stained with H&E to assess organization of the granulation tissue, anti-Ki-67 to analyze proliferation, and anti-PECAM-1 to assess vascular density of the sponge granulation tissue. SP = sponge matrix, arrows point at positive stain. (**B**) Proliferation graphed as number of Ki-67 positive cells/total tissue area and (**C**) vascular density graphed as percentage of immunopositive PECAM-1 area/total tissue area in histologic sections from granulation tissue. Data represents averages of multiple 40x fields from unpaired samples (*n* = 6). The statistical significance between experimental groups and control was determined by Mann Whitney Test, p<0.05 was considered statistically significant.

The PVA sponge model is used to study granulation tissue deposition that mimics healing by secondary intention [32], [33]. The effects of pyrvinium on granulation tissue organization, proliferation, and vascularization were analyzed and compared among the sponges implanted in multiple animals. Sponges injected with pyrvinium showed better granulation tissue organization when compared with its molecular analog, VU-WS211 (referred to as compd 211) (Figure 2A). The molecular analog of pyrvinium, compd 211, does not inhibits Wnt signaling [31]; hence used as a control. The tissue deposited within the sponges treated with compd 211 was less organized with poor architecture. The effect of pyrvinium-induced Wnt inhibition on cellular proliferation and tissue vascularity were assessed by anti-Ki-67 and anti-PECAM-1 staining, respectively. A significant increase in proliferation was evident in the sponges treated with pyrvinium (Figure 2A and 2B). In addition, anti-PECAM-1 immunostaining demonstrated that sponges treated with pyrvinium were better vascularized when compared with the sponges treated with compd 211 (Figure 2A and 2C). Taken together, these results demonstrate a positive correlation between pyrvinium treatment and tissue organization, proliferation, and vascularity during granulation tissue formation.

### Pyrvinium prevents adverse cardiac remodeling and increases proliferation in the injured myocardium

To evaluate pyrvinium-mediated myocardial regeneration, we assessed the therapeutic effect of pyrvinium in a mouse model of myocardial infarction. Myocardial infarcts were induced in mice by left coronary artery ligation, which produced infarcts in the anterolateral wall of the left ventricle (LV). Ten minutes after ligation, 200 nM of pyrvinium or compd 211 was injected at the junction of viable and infarcted tissue. Whereas no toxicity was observed when pyrvinium was injected subcutaneously into implanted sponges, a high number of mice (13 out of 22 animals) treated with intracardiac pyrvinium experienced lethal toxicity and died within the first 24 hours. Those that survived beyond the first 48 hours demonstrated similar activity and growth pattern as the control animals. Toxicity associated with pyrvinium was anticipated as IV or IP injection of pyrvinium at levels high enough to achieve Wnt-inhibitory plasma levels resulted in death within 24–48 hours in most mice (data not shown). This restricted our treatment regimen to a one-time intracardiac injection.

**Figure 3:**
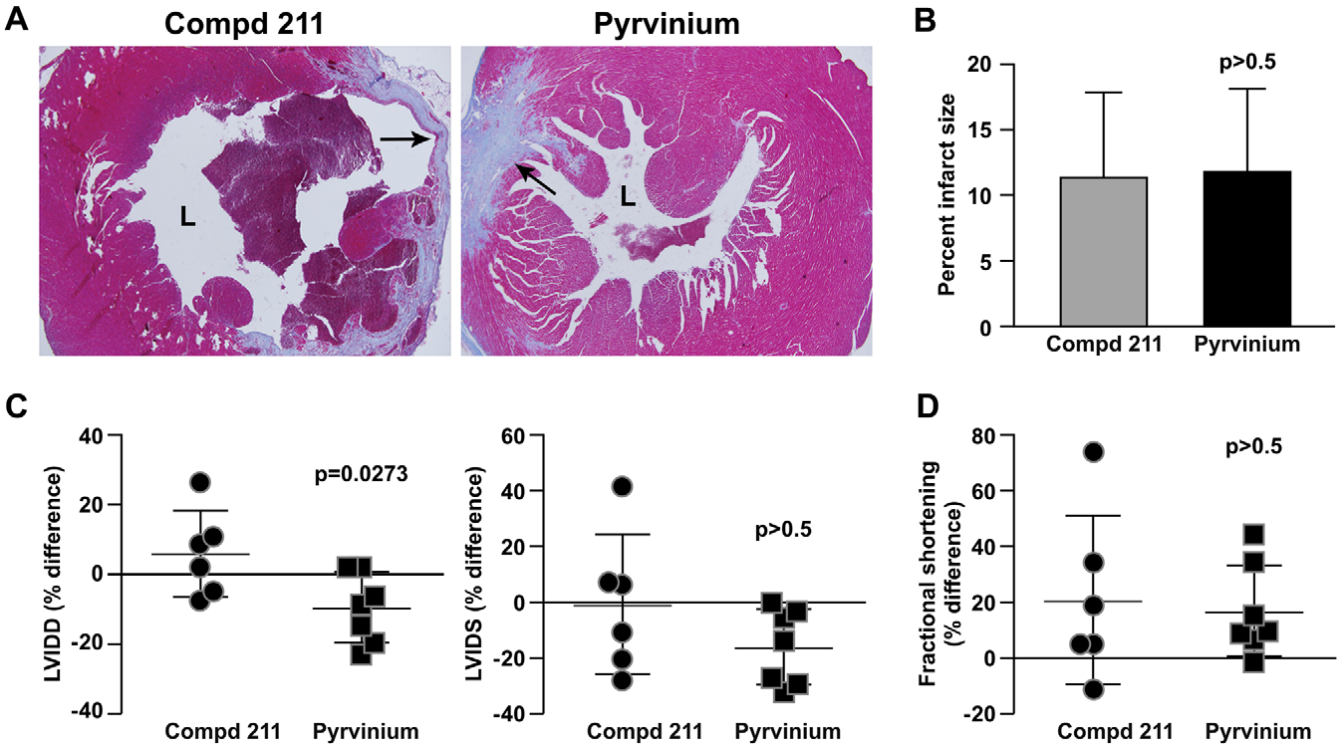
Pyrvinium prevents adverse myocardial remodeling. (**A**) Representative Masson trichrome-stained sections of hearts from mice 30 days after experimental infarct and treatment with pyrvinium and/or compd 211. Arrows point at the scarred ventricular wall. L = lumen. (**B**) The infarct size was quantified as the percentage of the left ventricular wall that exhibited myocyte replacement by scar. (**C**) LVIDD and LVIDS to represent cardiac remodeling, and (**D**) fractional shortening, as a measurement of cardiac function, were determined by echocardiography and are plotted as percentage difference values (mean +/− SEM) between 7 and 30 days after infarct. The statistical significance between experimental groups and control was determined by unpaired *t*-test (*n* = 6 for compd 211 and *n* = 7 for pyrvinium).

Masson trichrome-stained sections of hearts (Figure 3A) from mice 30 days after experimental infarct and treatment with pyrvinium or compd 211 showed no difference in the infarct size, as determined by the quantification of the scarred left ventricular wall (Figure 3B). Ventricular remodeling and cardiac function were analyzed by echocardiography at 7 and 30 days post MI. LV internal dimension diastolic (LVIDD) and LV internal dimension systolic (LVIDS) reflect remodeling of the infarcted ventricle (Figure 3C). The percentage difference in LVIDD between day 7 and day 30 was significantly reduced in animals receiving pyrvinium when compared with the control compd 211; −9.80±3.79 vs. 5.96±5.03 (p = 0.0273) (Table 1). Additionally, the percentage difference in LVIDS between day 7 and day 30 was reduced in animals treated with pyrvinium when compared with compd 211; −15.9±5.13 vs. −0.854±10.2; the data however was not statistically significant. Moreover, the percentage difference analysis of other echocardiogram parameters, such as interventricular wall thickness in diastole and systole (IVSD and IVSS), LV posterior wall thickness in systole (LVPWS) demonstrated a trend towards favorable heart remodeling (Figure S2 and Table 1). To determine whether pyrvinium improved cardiac function, the percentage differences in fractional shortening (FS) between 7 and 30 days after treatment were analyzed (Figure 3D). The average difference in fractional shortening of pyrvinium-treated vs. compd 211 control animals between days 7 and 30 was 16.9±6.19 vs. 20.9±12.4 (p>0.05). This observation, along with the absence of functional or anatomic difference between the two cohorts at day 7, provides greater evidence that pyrvinium did not acutely affect the extent of the infarct.

**Table 1:** Pyrvinium improves cardiac remodeling.

Echocardiogram parameters		Compd 211	Pyrvinium
**LVIDD, cm**	d7	0.341±0.0195	0.357±0.0162
	d30	0.356±0.00733	0.320±0.0133
**LVIDS, cm**	d7	0.218±0.0255	0.238±0.0170
	d30	0.205±0.00790	0.197±0.0140
**IVSD, cm**	d7	0.0723±0.00596	0.0779±0.00517
	d30	0.0768±0.00285	0.0737±0.00240
**IVSS, cm**	d7	0.106±0.00760	0.115±0.0119
	d30	0.118±0.00448	0.110±0.00480
**LVPWD, cm**	d7	0.0665±0.00437	0.0649±0.00550
	d30	0.0565±0.00426	0.0569±0.00152
**LVPWS, cm**	d7	0.0842±0.00333	0.088±0.00558
	d30	0.0887±0.00464	0.0761±0.00328
**FS**	d7	37.0±3.77	33.6±2.67
	d30	42.6±1.25	38.7±2.08
**EJ**	d7	0.726±0.0476	0.688±0.0353
	d30	0.799±0.0132	0.753±0.0275
**ΔLVIDD, %**		5.96±5.03	−9.80±3.79*
**ΔLVIDS, %**		−0.854±10.2	−15.9±5.13
**ΔFS, %**		20.9±12.4	16.9±6.19

The top eight rows represent the mean and standard error of mean (± SEM) values for each treatment at day 7 and day 30. The mean ± SEM percentage difference (Δ%) values are in the bottom three rows. The statistical significance between experimental groups and control was determined by unpaired *t*-test (*n* = 6 for compd 211 and *n* = 7 for pyrvinium).

*p<0.05 211 vs. pyrvinium.

**Figure 4:**
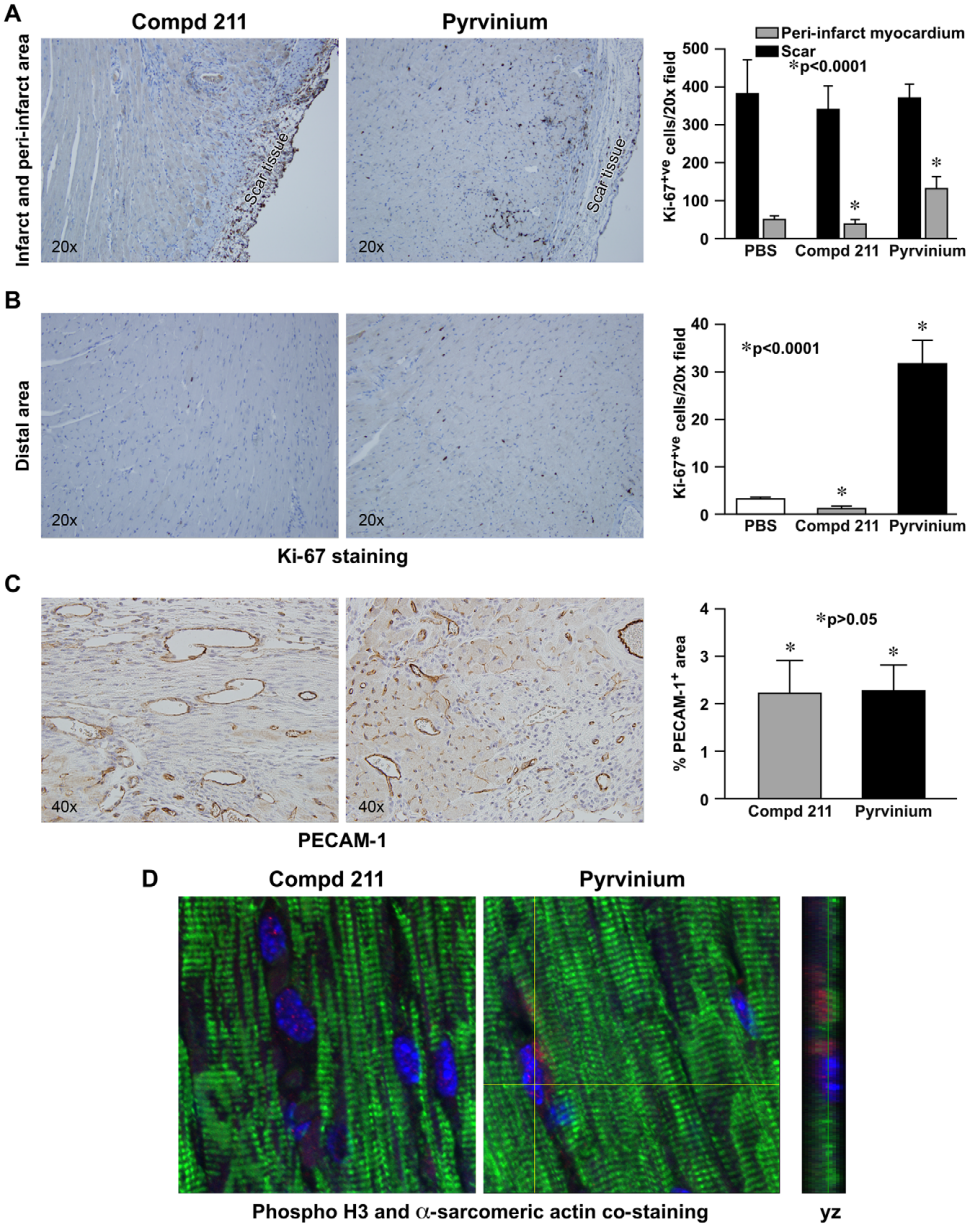
Pyrvinium promotes proliferation of myocytes in the peri-infarct and distal areas of the injured heart. (**A and B**) Representative images of anti-Ki-67 stained sections of compd 211- and pyrvinium-treated myocardium and the distal myocardium following MI. (**A and B**) Proliferation graphed as percentage of immunopositive Ki-67 positive cells/20x field in histologic sections from the proximal and the distal infarcted heart. (**C**) Representative images of anti-PECAM-1 staining to designate vascular density among experimental cohorts. Arrows point towards positive stain. Graph demonstrating the difference in PECAM-1 positive area/40x field. Data represents averages of multiple fields from unpaired samples (*n* = 6). (**D**) Representative immunostained confocal images of the sections of remote myocardium co-stained with anti-pH3 (1∶50; red) for cells undergoing mitosis, anti-alpha sarcomeric actin (1∶500; green) for cardiac muscles, and DAPI (blue) for the nuclei; yz axis designates anti-pH3 and anti-alpha sarcomeric actin co-staining. The statistical significance between experimental groups and control was determined by one way ANOVA with Newman-keuls post-test.

The identification both in genetic models [23], [24] as well as in our study that Wnt inhibition diminishes adverse post-infarct remodeling as well as the enhanced proliferation observed in sponge granulation tissue directed us to investigate if pyrvinium mediated Wnt inhibition can increase cardiomyocyte survival by enhancing cardiomyocyte proliferation. To assess proliferation, we analyzed the tissue with Ki-67, a nuclear protein necessary for cellular proliferation [34]. Pyrvinium treatment led to increased Ki-67^+^ staining of cells in the peri-infarct and distal myocardium (Figure 4A and 4B). Interestingly, vascular density within the myocardial scar, as identified by PECAM-1 staining, was not affected by pyrvinium treatment (Figure 4C). However, the numbers of active caspase-3^+^ cells, which reflect cells undergoing apoptosis, were not statistically different between pyrvinium and compd 211 treated myocardium (Figure S3).

We determined whether pyrvinium specifically increased cardiomyocyte mitosis utilizing an anti-phospho histone-3 antibody. Phosphorylation of histone 3 (pH3) on Ser 10 is an established cellular marker for chromosome condensation during mitotic prophase [35], [36]. We immunostained the distal myocardium using anti-pH3 and performed confocal microscopy to assess the effect of Wnt inhibition on the mitotic status of cardiomyocytes. pH3^+^ (red) cardiomyocytes exhibited a differentiated phenotype as indicated by striations and expression of α-sarcomeric actin (green) (Figure 4D). Reconstruction of optical sections enabled us to assign pH3^+^ nuclei unequivocally to cardiomyocytes (side panel). pH3^+^ cardiomyocytes were not evident in the remote myocardium of 211-treated hearts. These results indicate that a one-time pyrvinium injection is sufficient to increase proliferation of differentiated cardiomyocytes in the remote myocardium and promote favorable cardiac remodeling, albeit without a significant improvement in cardiac function or size of infarct.

## Discussion

Wnt signaling has been shown to be a major regulator of cardiogenesis [37], [38], [39]. Prior to gastrulation, Wnt/β-catenin signaling promotes cardiac differentiation whereas signaling during gastrulation inhibits heart formation [37], [38], [39]. Consistent with these studies, early treatment of mouse embryonic stem cells with Wnt3a stimulates mesoderm induction whereas late Wnt3a stimulation inhibits cardiac differentiation [40]. Furthermore, the Wnt inhibitors Dickkopf-1 (Dkk-1) and secreted frizzled-related proteins (sFRPs) have been shown to induce cardiac differentiation of stem cells [4], [37], [39]. These studies clearly demonstrate the importance of Wnt signaling in cardiac development. Additionally, gene expression profiling performed after myocardial infarct showed post-injury activation of Wnt signaling suggesting the role of Wnt signaling in cardiac repair [20].

Several antagonists of the Wnt pathways have been characterized [41]. One class, including sFRPs, binds and sequesters Wnt to inhibit both canonical and non-canonical Wnt signaling [41]. Fusion of Frizzled8-cysteine rich domain (binds Wnt) to the human Fc domain inhibited Wnt signaling and teratocarcinoma growth in mice but has not been widely used *in vivo* possibly due to its low *in vivo* efficacy or issues of selectivity [42]. The Dkk class inhibits canonical Wnt signaling by binding to LRP5/LRP6 of the Wnt receptor complex [41]. Recently a novel class of small molecule Wnt inhibitors has been identified that act by inhibiting tankyrase, a poly (ADP-ribose) polymerase [43], [44].

We recently have identified a FDA-approved drug, pyrvinium, as a potent inhibitor of Wnt signaling that acts by binding and activating CK1α [31]. Daily administration of pyrvinium by injection into PVA sponges, implanted subcutaneously, generated granulation tissue that was more cellular/proliferative, significantly better vascularized and with better tissue organization.

We further assessed if pyrvinium can be used as a therapeutic Wnt inhibitor in cardiac injury. Pyrvinium-treated mice demonstrated significantly more favorable LV remodeling 30 days post-MI compared to a control compound. Cardiac remodeling is a physiological response and a compensatory change in the structure and function of the heart in response to cardiac injury or other pathological conditions. Although, early remodeling is an adaptive response to maintain cardiac function, it progressively becomes maladaptive and results in the progression of cardiovascular diseases. Therefore, identification of a therapeutic inhibitor to help prevent the process of adverse cardiac remodeling is highly desirable. Remarkably, peri-infarct intramuscular administration of pyrvinium prevented adverse cardiac remodeling and this effect was observed after only a single dose of intramuscular administration of the Wnt inhibitor following injury. Because we were limited to a single intramuscular administration of pyrvinium after injury, we are unable to confidently assess the effect of Wnt inhibition on cardiac function/infarct size. Nonetheless, mice that survived after the pyrvinium treatment showed favorable cardiac remodeling after MI, indicating the efficacy of a pharmaceutical Wnt inhibitor in the prevention of adverse cardiac remodeling.

To better understand the basis for how therapeutic Wnt inhibition might affect cardiac remodeling, we tested whether pyrvinium affected cellular apoptosis in both PVA granulation tissue and in infarcted myocardium. We did not observe any statistically significant differences in level of cellular apoptosis by analyzing the numbers of activated caspase-3 positive cells in pyrvinium treated animals as compared to those treated with control compound (Figure S3). Prevention of cell loss by apoptosis as a result of inhibition of caspase activation through therapeutic interventions can protect cardiac function [45]. In myocardial ischemia/reperfusion-induced injury the non-specific inhibition of caspases may be of therapeutic benefit [46]. Multiple *in vivo* and *ex vivo* models have correlated the quantity of apoptotic cell numbers with the cardiac function and infarct size indicating the significance of apoptosis during and after cardiac injury [47], [48]. Although determination of absolute values of apoptosis would require the combination of several techniques and limiting analysis of myocardial tissue at 30 days post infarct may have missed any short term effects on apoptosis, these data suggest that apoptosis is unlikely to account for the observed phenotype.

In granulation tissue formation within both PVA sponges and infarcted hearts, Ki-67^+^ cells were significantly increased with pyrvinium treatment. Increased cardiomyocyte DNA synthesis evident by Ki-67^+^ cells in both the border zone and remote area suggested that some differentiated cardiomyocytes re-enter the cell cycle, which we further confirmed by co-localizing a mitotic marker (anti-pH3) with a myocyte protein. Interestingly, we did not observed any difference in the vascularization of the infarcted heart treated with pyrvinium when compared with the control compd 211. This observation supports our conclusion that increased proliferation in pyrvinium-treated heart cells is not due to better vascularization but due to direct inhibition of Wnt signaling in cardiomyocytes. While we have shown that a subpopulation of differentiated cardiomyocytes has proliferative potential, our results do not exclude the possibility that pyrvinium-mediated Wnt inhibition may have multifactorial impact that contribute to the observed favorable post-injury global remodeling.

The exploitation of small molecule Wnt inhibitors would facilitate the development of therapeutic reagents. Our study indicated that a pharmacologic Wnt inhibitor may be a promising tool to promote tissue repair and prevent adverse cardiac remodeling. Understanding the therapeutic value of Wnt inhibition in cardiac injury using pyrvinium is limited by its toxicity. Yet, the basis for pyrvinium's toxicity, as well as that of other small molecular Wnt inhibitors is not clearly established. Pyrvinium regulates Wnt signaling by activating CK1α and regulating the stabilization of β-catenin and Axin in the cytoplasm. The CK1α family of serine/threonine kinases is evolutionarily conserved in eukaryotes and is associated with a wide range of cellular processes that includes cell cycle, apoptosis, and Wnt signaling [49]. It is not clear whether the toxicity that is associated with pyrvinium is due to its effects on CK1α or to its potential alkylating activity (data not shown). Nonetheless, our studies have demonstrated the possibility of utilizing a small molecule Wnt inhibitor as a curative agent due to its ability to positively affect wound repair and regeneration both *in vitro* and *in vivo*. Therefore, despite the limitations resulting from *in vivo* toxicity, these findings highlight the potential of Wnt inhibition to treat MI and the need for a safe and effective therapeutic Wnt inhibitor to better dissect the effect of Wnt inhibition on cardiac repair and regeneration. Our ongoing studies are to characterize newly identified small molecule Wnt inhibitors as well as antibody based inhibitors to better define and understand the mechanistic basis for adverse effects of systemic Wnt inhibition. Identification of a non-toxic Wnt inhibitor will enable us to more rigorously test the utility of Wnt inhibitors as therapeutic agents to enhance repair and regeneration.

## Materials and Methods

### Animals

This study was carried out in strict accordance with the recommendations in the Guide for the Care and Use of Laboratory Animals of the National Institutes of Health. The protocol was approved by the Vanderbilt University Institutional Animal Care and Use Committee (Protocol Number: M/07/236). All experiments were performed using appropriate analgesics and anesthetics, and every effort was made to minimize pain/distress.

### Antibodies and Reagents

Antibodies against specific antigens were purchased as following: CD31/platelet endothelial cell adhesion molecule-1 (PECAM-1; clone 557355, PharMingen), Ki-67 (Novocastra Labs, NCLKi67p), phospho histone-3 (Millipore). α-sarcomeric actin (Sigma), β-catenin (BD Transduction Labs), Axin1 (R&D Antibodies), β-tubulin (Santa Cruz), HA 3F10 (Roche), and β-galactosidase (Promega). Appropriate secondary antibodies were purchased from Jackson ImmunoResearch, Santa Cruz, and Molecular Probes. 4',6-diamidino-2-phenylindole (DAPI) was purchased from Vector laboratories Inc. Recombinant Wnt3a was purchased from (R&D). Recombinant human CK1α and GSK3 were purchased form Invitrogen. Casein was purchased from Sigma (C8032). Pyrvinium chloride and VU-WS211 were synthesized by the Vanderbilt Institute of Chemical Biology's medicinal chemistry core. DMSO was used as a vehicle for pyrvinium in all experiments unless otherwise stated.

### Cell lines

HEK 293 STF was a gift from Jeremy Nathans. Wnt3a cells were purchased from ATCC. Cells were maintained in DMEM, 10% fetal bovine serum (FBS), and antibiotics. Cells were treated with compounds and/or Wnt for 24 hours unless otherwise stated.

### Reporter assays

For cell-based luciferase assays, HEK 293 STF cells were seeded into 96-well plates at sub-confluent levels and luciferase activities measured by Steady-Glo Luciferase Assay (Promega). Luciferase activities were normalized to viable cell number using the CellTiter-Glo Assay (Promega). All graphs were made in Prism 4 (GraphPad Software, inc.) with nonlinear regression fit to a sigmoidal dose-response curve (variable slope). Wnt3a and pyrvinium were added 24 hours after transfection for an additional 24 hours.

### Dot blot and kinase assay

For ligand dot blot assay, purified proteins CK1α and GSK3 (0.5 ug protein each) were dotted on nitrocellulose membranes and blocked for 1 hour using 5% milk in TBS. Pyrvinium was then added and incubated for 3 hours at 23°C. Membrane was then washed three times for 5 minutes in TBS plus 0.1% Tween-20. The pyrvinium fluorescence image was acquired on a Xenogen IVIS 200 using excitation 500-550 and emission 575-650 spectrum fluorescence settings. *In vitro* kinase assay was performed as previously described [29].

### RNA isolation, cDNA synthesis, and real-time PCR

Total RNA was isolated from HEK 293 cells 24 hours after pyrvinium treatment using RNAeasy RNA extraction kit (Qiagen), and cDNA generated using High Capacity cDNA Reverse Transcription kit (Applied Biosystems, ABI). Real-time PCR assays were performed in quadruplicate using TaqMan Gene Expression Master Mix (ABI), gene-specific TaqMan TAMRA probes (ABI) and an ABI 7000 sequence detection system.

### Repair/Granulation Tissue Stimulation

For repair/granulation tissue stimulation model, PVA sponge discs (4 mg, 2 mm height, 4 mm diameter) were presoaked with 40 µl of matrigel and implanted subcutaneously in adult mice. Two sponges were implanted in each mouse and three mice were used per treatment. Following ten days of sponge implantation mice were injected with either 200 nM pyrvinium or compd 211 (in 40 µl PBS per injection) every day or every other day, receiving nine injections in total. Twenty one day after sponge implantation animals were taken down. The sponges were fixed in 10% neutral buffered formalin and stored at −80°C until use.

### Myocardial Infarction

For MI model, C57Bl6 mice were anesthetized with sodium pentathol (50 mg/kg) and endotracheal intubation was performed under direct laryngoscopy. Mice were ventilated with a small animal respirator (tidal volume  = 1.0 ml, rate  = 110 breaths/min). With the use of a surgical microscope, a left thoracotomy was performed. The fourth intercostal space was entered using scissors and blunt dissection. A 7-0 silk suture was placed through the myocardium into anterolateral LV wall (around the left anterior descending artery) and the artery ligated. Continuous EKGs were obtained during the procedure. 200 nM of pyrvinium or compd 211 in 25 µL of PBS were injected into the peri-infarct area. The chest was closed in layers with 6-0 silk (6-0 nylon to close the skin) and the animal was gradually weaned from the respirator to avoid complicating pneumothorax. Cardiac dimensions were obtained from 2-D guided M-mode images (100 frames/sec) and were read blinded using short axis and a parasternal long-axis views with the leading edge method. All measurements were done on unsedated mice. Measurements were averaged over 3 consecutive beats from the LV posterior wall (LVPW) the interventricular septum (IVS) and LV internal diameter (LVID). Fractional shortening (FS) and ejection fraction (EF) were obtained at day 7 and day 30 post MI. Excised hearts were immersion-fixed in 10% buffered formalin for 24 hours and transferred to 70% ethanol to obtain serial sections in order to measure the infarct size. Subsequently, serial sections through the ventricles were obtained parallel to the atrioventricular groove and the samples were processed for light microscopy. Paraffin sections were stained with H & E and Masson trichrome. In order to measure the infarcted areas in all sections on trichrome-stained slides, the percentage of left ventricle that exhibits myocyte replacement by scar was quantified using Image Pro software (Media Cybernetics) [50].

### Histochemistry and Morphometry

PVA Sponges were embedded with cut surface down for histology. Immunohistochemistry for PECAM-1 to analyze vascularity and Ki-67 to identify proliferation was performed as described by Young et al [51], [52]. A CoolSNAP Hq CCD camera (Photometrics) was utilized to obtain the images of PECAM-1 stained sections. Around 10 digital images from each section were acquired at defined magnification (40×) at random for vascular density. The area of tissue for each field was quantified using MetaMorph (Molecular Devices) by outlining tissue and calculating total area per field. For Ki-67 analysis, around 10 digital images were taken at random from each section at specific magnification (40×). The images were acquired with a digital camera (Pixera, Los Gatos, CA). and positively stained cells were counted manually.

### Confocal Microscopy

Paraffin-embedded cardiac tissue slides were deparaffinized, blocked in 10% goat serum for one hour and co-stained with anti-alpha sarcomeric actin and phospho-histone-3 antibodies overnight at 4°C. The slides were washed in PBS and co-stained with goat anti-mouse and goat anti-rabbit secondary antibodies for two hours, washed in PBS, and mounted with DAPI. For confocal analysis, LSM510 (Zeiss) microscope was used to capture 1 µm optical slices (z stack); the images were analyzed with Metamorph v5.0 (Universal Imaging Corp.).

### Statistical Analysis

The statistical significance between experimental groups and control was determined by unpaired Student's *t*-test, Mann Whitney Test, or ANOVA followed by Newman-Keuls post- test as designated using GraphPad Prism. p<0.05 was considered statistically significant.

## Supporting Information

Figure S1 **Pyrvinium inhibits Wnt signaling.** (**A and B**) Pyrvinium decreases and increases intracellular β-catenin (*, **p<0.005, *t*-test) and Axin (*p<0.05, **p<0.005, *t-*test) levels, respectively. HEK 293 cells were treated for 16 hours as indicated, and cytoplasmic preparations were immunoblotted for β-catenin and Axin. Quantification of the relative cytoplasmic β-catenin protein levels normalized to β-tubulin; n = 5. (**C**) Pyrvinium decrease steady-state levels of Pygopus. HEK 293 STF cells expressing HA-tagged human Pygopus-2 were treated with pyrvinium as indicated. Lysates were immunoblotted for HA. Quantification of the relative pygopus levels normalized to β-galactosidase (β-gal) (*, **p<0.005, *t-*test); n = 5. Quantitation of immunoblots were performed by scanning images with Adobe Photoshop CS4 (Adobe Systems) and the intensity of the bands quantified with NIH Image J with correction for background. (**D, E, and F**) Pyrvinium (100 nM) decreases transcript levels of endogenous Wnt target genes Myc, Dkk-1, and Axin2 as assessed by real-time PCR. Relative transcript levels normalized to GAPDH (*p<0.05, **p<0.005, *t*-test); n = 3.
(TIF)

Figure S2 **Pyrvinium prevents adverse myocardial remodeling.** LVPWS, LVPWD, IVSD, and IVSS to represent cardiac remodeling, and ejection fraction, as a measurement of cardiac function, were determined by echocardiography and are plotted as percentage difference values (mean +/− SD) between 7 and 30 days after infarct. The statistical significance between experimental groups and control was determined by unpaired *t*-test.
(TIF)

Figure S3 **Pyrvinium treatment does not affect cellular apoptosis.** Representative images of the pyrvinium- and compd 211-treated sponges stained with anti-caspase-3 and histological sections of anti-caspase-3 stained compd 211- and pyrvinium-treated myocardium following MI. SP = sponge matrix, arrows point at positive stain.
(TIF)
