# Supplementary figures and images for: Pyrvinium, a Potent Small Molecule Wnt Inhibitor, Promotes Wound Repair and Post-MI Cardiac Remodeling
Source: PLoS One. 2010 Nov 29;5(11):e15521. doi: 10.1371/journal.pone.0015521 (PMC2993965; doi:10.1371/journal.pone.0015521)

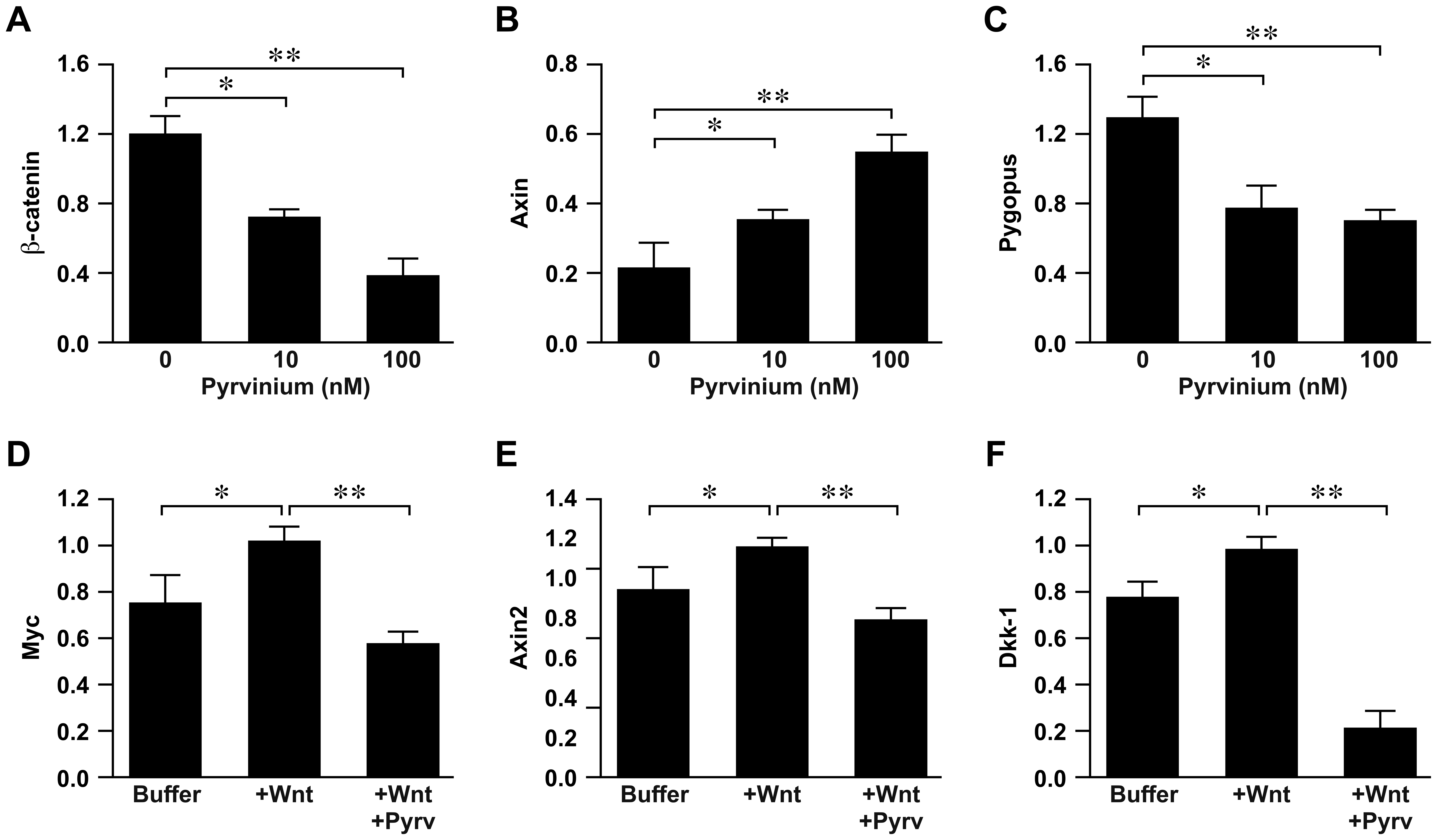

Supplement: Figure S1 — Pyrvinium inhibits Wnt signaling. (A and B) Pyrvinium decreases and increases intracellular β-catenin (*, **p<0.005, t-test) and Axin (*p<0.05, **p<0.005, t-test) levels, respectively. HEK 293 cells were treated for 16 hours as indicated, and cytoplasmic preparations were immunoblotted for β-catenin and Axin. Quantification of the relative cytoplasmic β-catenin protein levels normalized to β-tubulin; n = 5. (C) Pyrvinium decrease steady-state levels of Pygopus. HEK 293 STF cells expressing HA-tagged human Pygopus-2 were treated with pyrvinium as indicated. Lysates were immunoblotted for HA. Quantification of the relative pygopus levels normalized to β-galactosidase (β-gal) (*, **p<0.005, t-test); n = 5. Quantitation of immunoblots were performed by scanning images with Adobe Photoshop CS4 (Adobe Systems) and the intensity of the bands quantified with NIH Image J with correction for background. (D, E, and F) Pyrvinium (100 nM) decreases transcript levels of endogenous Wnt target genes Myc, Dkk-1, and Axin2 as assessed by real-time PCR. Relative transcript levels normalized to GAPDH (*p<0.05, **p<0.005, t-test); n = 3. (TIF) [file pone.0015521.s001.tif]

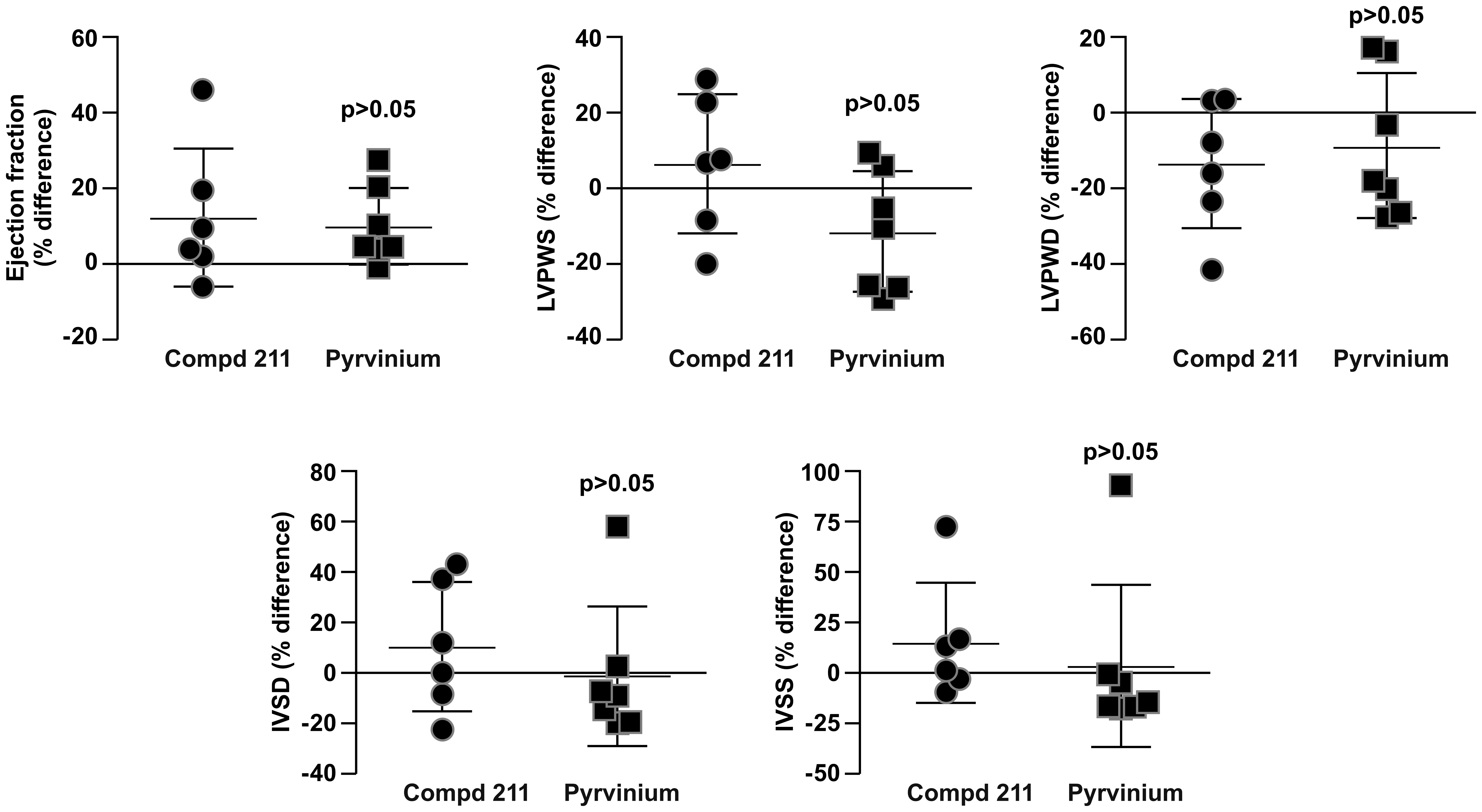

Supplement: Figure S2 — Pyrvinium prevents adverse myocardial remodeling. LVPWS, LVPWD, IVSD, and IVSS to represent cardiac remodeling, and ejection fraction, as a measurement of cardiac function, were determined by echocardiography and are plotted as percentage difference values (mean +/− SD) between 7 and 30 days after infarct. The statistical significance between experimental groups and control was determined by unpaired t-test. (TIF) [file pone.0015521.s002.tif]

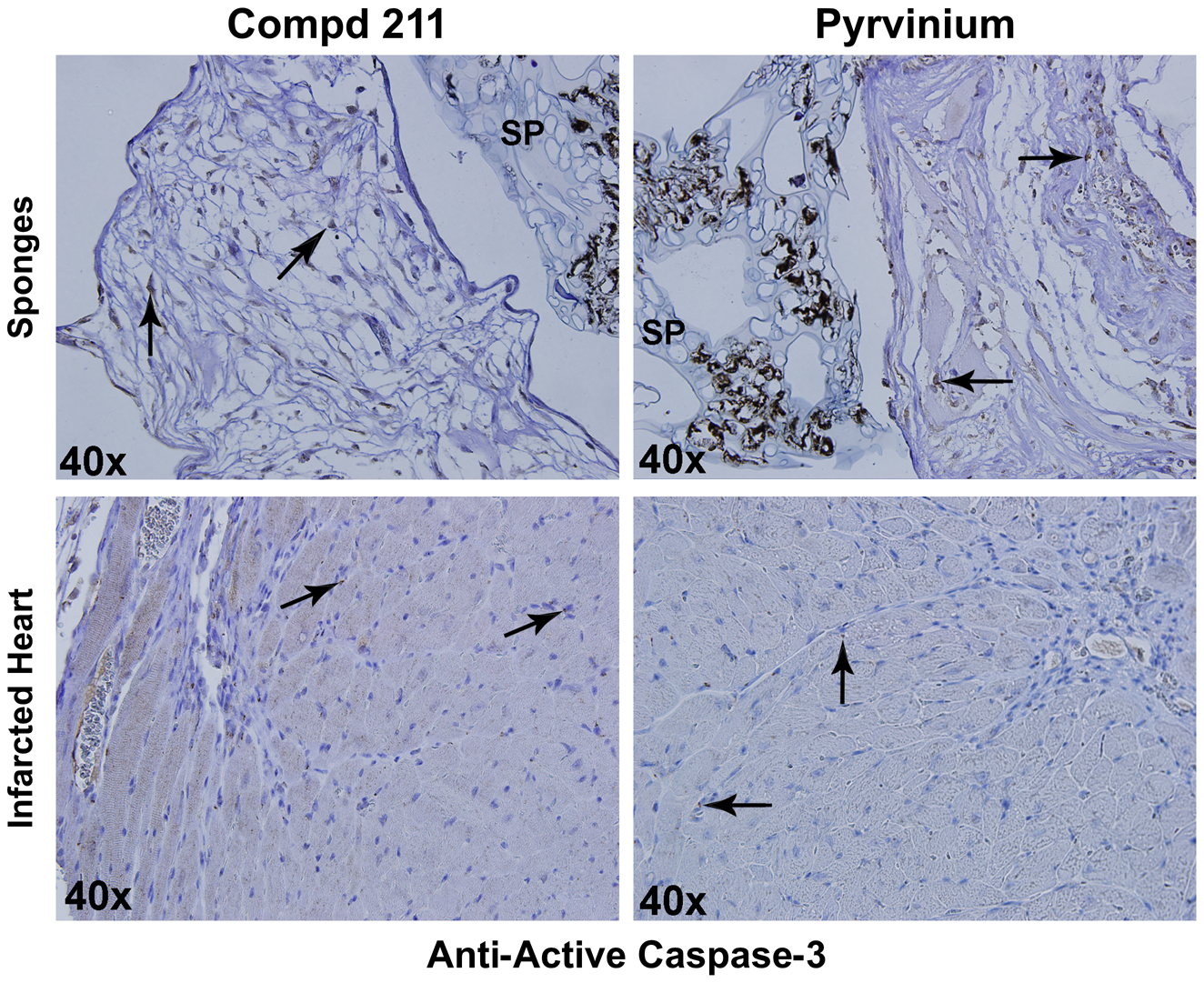

Supplement: Figure S3 — Pyrvinium treatment does not affect cellular apoptosis. Representative images of the pyrvinium- and compd 211-treated sponges stained with anti-caspase-3 and histological sections of anti-caspase-3 stained compd 211- and pyrvinium-treated myocardium following MI. SP = sponge matrix, arrows point at positive stain. (TIF) [file pone.0015521.s003.tif]
